# Wombat Roadkill Was Not Reduced by a Virtual Fence. Comment on Stannard et al. Can Virtual Fences Reduce Wombat Road Mortalities? *Ecol. Eng.* 2021, *172*, 106414

**DOI:** 10.3390/ani12101323

**Published:** 2022-05-23

**Authors:** Graeme Coulson, Helena Bender

**Affiliations:** 1 School of BioSciences, University of Melbourne, Parkville, VIC 30101, Australia; 2 School of Ecosystem and Forest Sciences, University of Melbourne, Parkville, VIC 3010, Australia; hbender@unimelb.edu.au

**Keywords:** bare-nosed wombat, roadkill, vehicular traffic, virtual fence, *Vombatus ursinus*, wildlife-vehicle collisions

## Abstract

**Simple Summary:**

Wildlife roadkill is a global problem. Devices that produce bright lights and loud noises are claimed to be effective deterrents, but there is no scientific evidence that they actually prevent collisions between vehicles and wildlife. A recent trial in Australia installed a light and sound system, marketed as a ‘virtual fence’, and tested it on bare-nosed wombats (*Vombatus ursinus*). The study concluded that the system was minimally effective but promising. We detected a number of conceptual and procedural flaws in that study. When we re-analysed their data, we found absolutely no evidence for the effect of the virtual fence on the roadkills of wombats.

**Abstract:**

The roadkill of wildlife is a global problem. Much has been written about deterring wildlife from roads, but, as of yet, there is no empirical support for deterrents based on visual and/or auditory signals. A recent paper entitled ‘Can virtual fences reduce wombat road mortalities?’reported the results of a roadkill mitigation trial. The authors installed a ‘virtual fence’ system produced by iPTE Traffic Solutions Ltd. (Graz, Austria) and evaluated its effectiveness for reducing roadkills of bare-nosed wombats (*Vombatus ursinus*) in southern Australia. The authors recorded roadkills in a simple Before-After-Control-Impact design but did not conduct any formal statistical analysis. They also measured three contextual variables (vegetation, wombat burrows, and vehicle velocity) but did not link these to the occurrence of roadkills in space and time. The authors concluded that the iPTE virtual fence system was ‘minimally effective’, yet ‘appears promising’. Our analysis of their data, using standard inferential statistics, showed no effect of the virtual fence on roadkills whatsoever. We conclude that the iPTE system was not effective for mitigating the roadkills of bare-nosed wombats.

## 1. Introduction

Wildlife-vehicle collisions, commonly known as roadkills, are a serious global problem: animal welfare is impacted, population viability is threatened, human safety and amenity are diminished, and vehicle damage is costly [1,2,3]. Identifying strategies for mitigating roadkill is, therefore, important for living harmoniously with wildlife, and many mitigation methods have been devised [4,5]. However, progress in the field of roadkill mitigation has been impeded by poor experimental design in many studies [4,5,6].

In a recent paper in the journal Ecological Engineering, Stannard et al. [7] reported the findings of a trial of a virtual fence that was installed to mitigate the roadkills of bare-nosed wombats (*Vombatus ursinus*) on a rural road in southern New South Wales, Australia. Stannard et al. [7] concluded that the virtual fence ‘appeared to be minimally effective at reducing roadkill’ of bare-nosed wombats, yet ‘virtual fencing appears promising’. In this Commentary, we evaluate each component of the study by Stannard et al. [7]. We identify a number of weaknesses in the framing and design of their study, as well as in their analysis and interpretation of the data. We then reappraise their conclusions.

## 2. Conceptual Framework

Stannard et al. [7] framed their study within the field of virtual fencing, which is defined as ‘a structure serving as an enclosure, a barrier, or a boundary without a physical barrier’ [8]. The fence tested by Stannard et al. [7] is produced by iPTE Traffic Solutions Ltd. (Mantscha-Wald-Weg 48, 8054 Graz, Austria) and marketed in Australia by Wildlife Safety Solutions Pty Ltd. (8 Arden Street, Waverley, New South Wales 2024, Australia). The Wildlife Safety Solutions fence comprises a set of electronic units spaced alternately along the roadside, emitting sound and light stimuli when activated by the headlights of approaching vehicles [9]. As such, this fence conforms to the definition of virtual fencing in the broad sense [8] but is an outlier. Virtual fencing systems are typically animal-borne and are used to control the movement of companion animals [10,11] and livestock [12,13], not wildlife.

The Wildlife Safety Solutions system tested by Stannard et al. [7] takes a different approach to the animal-borne systems instead of using stand-alone devices that produce audio and visual signals. These signals are directed towards wildlife habitats bordering the road, with the aim of deterring the animals from entering the roadway when a vehicle is approaching [9]. As the Wildlife Safety Solutions system is targeted at wild animals near roads, the framing of the study by Stannard et al. [7] is misplaced. A more appropriate context would have been the field of roadkill mitigation by deterrence or exclusion of wildlife. Much has been written about deterring wildlife from roads, but few methods have proved to be effective [4]. As yet, there is no support for the effectiveness of deterrents based on either visual [14,15,16] or auditory [17,18,19] signals. There is also no support so far for deterrent systems that use a combination of visual and auditory signals [20]. Indeed, visual and auditory signals are not likely to be effective deterrents: the wavelength and intensity are unlikely to be suited to field conditions, and wildlife on or near the road may not appropriately respond even if they can detect the warning signal [21].

Given the lack of empirical support for visual and auditory deterrents on roadsides, Stannard et al. [7] needed to make a solid case for testing the Wildlife Safety Solutions system. This system does have some novel features, including flashing LEDs in two colours interspersed with an acoustic signal, which are all actuated by vehicle headlights [9]. However, the authors did not consider the visual and auditory capabilities of wombats or consider the appropriate behavioural responses that wombats might display. Instead, the authors drew, uncritically and erroneously, on the two field studies conducted in Tasmania, Australia. The authors cited a study [22] that reported a 50% reduction in the rate of wildlife killed on the road after the fence system was installed. However, Stannard et al. [7] did not recognise the limitations of the study’s design nor acknowledge that the analyses and conclusions of this study were contested [23,24]. Stannard et al. [7] cited another study [25], claiming that the fence system reduced roadkill rates by 21% to 57%, depending on the species. However, this statement completely misrepresents the findings of the second study, which concluded that the virtual fence had no detectable effect on the roadkill rate [25]. That study used a sophisticated Crossover and Multiple Before-After-Control-Impact (BACI) design, as well as a Generalised Additive Model to adjust for the spatial and temporal change and a simple fence on-off comparison, but found no fence effect in any of these complementary analyses [25]. In that study, it was acknowledged that the statistical power of the analyses constrained their sensitivity to detect any reduction in roadkill rate, such that they could detect a 50% reduction but not a 20% reduction in roadkills [25]. The 21–57% reductions claimed by Stannard et al. [7] were merely the simulated reductions used in the power analysis in the second study [25] (Table 1, not their observed changes in roadkill rate. The portrayal of these two studies is therefore misleading at best and offers little support for the application of this fence system to mitigate the roadkill of wombats.

## 3. Experimental Design, Materials and Methods

### 3.1. Study Design

Stannard et al. [7] adopted a basic *before-after-control-impact* (BACI) design, although they did not refer to it as such. They monitored wombat roadkills along a 22.7-km section of road beginning in June 2017 *before* the virtual fence was installed on a 1.5-km sub-section of the same road in March 2020. The authors then monitored a short, 1.5-km *impact* section and the remaining 21.1-km *control* length of that road for 11 months *after* the installation of the fence. The authors stated that they selected their study on the basis of roadkill records in the WomSAT database (womsat.org.au, accessed on 20 March 2022) but did not explain how these data informed their choice of the *impact* (virtual fence) or *control* sections. No other justification for site selection was given. The authors described the road as a single carriageway in a rural area, with a speed limit of 100 km/h, but did not mention spatial characteristics of the road, such as horizontal and vertical curvature, which are known to influence the probability of roadkills of wildlife [26], including the study species, the bare-nosed wombat [27]. It is also unclear whether the study site is typical of areas with wombat roadkills [27,28], so the generality of the findings is unknown.

### 3.2. Data Aquisition

To record wombat roadkills as they occurred during their study, Stannard et al. [7] relied on the WomSAT database (womsat.org.au, accessed on 20 March 2022). The authors did not specify whether they simply used WomSAT as a recording tool or whether records were uploaded by citizen scientists. If the former, the authors give no indication of the locational accuracy of the WomSAT system, which enables users to upload data via a mobile phone app or a web browser on their computer [29]. The quality of the data, therefore, depends on the locational accuracy of the user’s mobile phone, which is generally inferior to a hand-held GPS unit [30,31], or their proficiency in using the mapping tool in the web-based version. These limitations would also apply to data contributed by citizen scientists, whose involvement raises additional concerns about data quality, which pervade the field of citizen science [32,33]. Crucially, Stannard et al. [7] did not specify whether the roadkill records could be confidently allocated to the *treatment* or *control* sections of the road.

### 3.3. Context Variables

Stannard et al. [7] measured three context variables: vegetation, wombat burrows and bolt holes, and traffic velocity. However, these were measured only in the *impact* section of the road; the absence of comparable data from the *control* section prevented any meaningful comparison of these three variables over the course of the study. Furthermore, the authors failed to establish the relevance of these variables to their evaluation of the virtual fence.

#### 3.3.1. Vegetation Survey

Stannard et al. [7] measured the structure and composition of trees, shrubs and ground cover along 4-m wide and 25-m long belt transects, placed perpendicular to the road at 150-m intervals on alternating sides of the road. No explanation was offered for the length and width of the transect or the spacing interval, and the date of the survey was not specified.

#### 3.3.2. Burrow Survey

Stannard et al. [7] recorded wombat burrows and bolt holes along the transects without explaining the significance of the distinction drawn between these structures. Furthermore, the authors did not elucidate how vegetation and burrows within only 25 m of the road might be linked to the occurrence of roadkills, given the scale of wombat movements. Although estimates vary, bare-nosed wombats have home ranges covering at least 2 ha, sometimes exceeding 1000 ha, and individuals can move > 6 km in one night [34], suggesting the scale of the burrow survey is unlikely to provide useful insights.

#### 3.3.3. Traffic Velocity

To measure the traffic velocity, Stannard et al. [7] installed Viasis 3003 speed indicator signs (Via Traffic Controlling, Germany) at each end of the *impact* section for the first two months of the *after* phase. The Viasis device uses continuous Doppler radar to measure vehicle velocity, which is then displayed publicly on an LED screen and stored for later download. However, the authors did not specify whether the display was active, thereby giving drivers feedback on their speed and likely influencing their subsequent behaviour [35] as they entered the *impact* section, generating a potentially serious confounding variable.

## 4. Results and Discussion

Stannard et al. [7] used Analysis of Variance (ANOVA) to show that tree canopy cover and shrub cover was unevenly distributed along the margins of the *impact* section of road, whereas they found no difference in percentage ground cover or dominant species. These findings simply reflected the heterogeneity of the site, which had remnant bushland concentrated at the eastern end and southern side of the road. Nonetheless, the authors concluded from these data that the habitat along the roadside was ‘ideal for wombats’. The authors also recorded 31 burrows, of which seven had signs of recent use, as well as six bolt holes, but the relevance of these observations was not made clear.

Stannard et al. [7] reported the vehicle velocity at each end of the *impact* section, expressed as mean, maximum, and ‘85% of the time’, presumably the 85th percentile. However, the authors did not report any data on the volume of traffic, despite its well-known influence on the probability of wildlife roadkill [36], including the study species [37].

Stannard et al. [7] reported the raw frequencies of wombat roadkills *before* and *after* the fence was installed and in the *control* and *impact* sections of the road. The authors also reported the rate of wombat roadkills per month, confusingly referred to as a ‘ratio’ when two different units of measurement (roadkill frequency and time) were involved. Inexplicably, the authors did not conduct any formal statistical analysis of these data. We analysed their raw data as a simple Chi-square 2 × 2 contingency table (Table 1). There was no association whatsoever between time (*before* or *after*) and treatment (*control* or *impact*): *X^2^* = 0.0011, df = 1, *p* = 0.97299.

## 5. Conclusions

From their vegetation survey, Stannard et al. [7] concluded that the roadside habitat in their study site provided a ‘suitable location for burrowing and foraging’, with ‘wombats actively living in the area’. This was self-evident given the occurrence of roadkills. Surprisingly, although the authors collected these data on vegetation structure and composition, and complementary burrow data, they made no attempt to relate these biotic variables to the distribution or abundance of roadkills. Habitat is a factor known to influence the probability of roadkills of the bare-nosed wombat [28], as it does in many other wildlife species [38,39].

From their traffic survey, Stannard [7] noted that vehicle speeds were higher at one end of the *impact* section of the road than at the other. They did not, however, relate traffic speed to the distribution or abundance of roadkills, despite studies showing the influence of vehicle speed on the risk of becoming roadkill for many wildlife species [40,41], amongst them the bare-nosed wombat [37]. Stannard et al. did consider vehicle speed in terms of the distance at which wildlife can be detected on the road at night, noting that the mean vehicle speeds recorded on the impact section were within the range that allowed drivers time to detect animals on the road [42]. However, this discussion was irrelevant, as was the speculation about the detectability of varied coat colours in wombats; if the virtual fence functioned as claimed, any wombats nearby should have been deterred from approaching, so drivers would not need to detect them.

In their Discussion, Stannard et al. [7] twice described the virtual fence as ‘minimally effective’ at reducing roadkills of bare-nosed wombats. The authors expressed the rate of wombat roadkills per month *after* fence installation as ‘almost the same’ and ‘only slightly lower’ than *before* the installation. They referred to their ‘preliminary findings’ and argued that ‘further data are needed’. Had the authors made proper use of their informal BACI design, and used robust, inferential statistics, they could only have concluded that there was no statistically significant effect.

Stannard et al. [7] posed a simple question in their title: ‘Can virtual fences reduce wombat road mortalities?’ From the data presented, the unequivocal answer is no.

## Figures and Tables

**Table 1:** Observed frequency of roadkills of bare-nosed wombats (*Vombatus ursinus*) in the *control* and *impact* road sections *before* and *after* installation of a virtual fence. Data from Table 1, Supplementary Data of Stannard et al. [7]. Expected frequencies (in italics) from the null hypothesis of no association between treatment (*control/impact*) and time (*before/after*).

	Before	After	Total
**Control**	64	17	81
	*64.06*	*16.94*	
**Impact**	23	6	29
	*22.94*	*6.06*	
**Total**	87	23	110

## Data Availability

Not applicable.

## References

[1] Englefield B., Starling M., McGreevy P. (2018). A review of roadkill rescue: Who cares for the mental, physical and financial welfare of Australian wildlife carers?. Wildl. Res..

[2] Bíl M., Andrášik R., Duľa M., Sedoník J. (2019). On reliable identification of factors influencing wildlife-vehicle collisions along roads. J. Environ. Manag..

[3] Abraham J.O., Mumma M.A. (2021). Elevated wildlife-vehicle collision rates during the COVID-19 pandemic. Sci. Rep..

[4] Rytwinski T., Soanes K., Jaeger J.A.G., Fahrig L., Findlay C.S., Houlahan J., van der Ree R., van der Grift E.A. (2016). How effective is road mitigation at reducing road-kill? A meta-analysis. PLoS ONE.

[5] van der Grift E.A., Findlay S., van der Ree R., Fahrig L., Houlahan J., Jaeger J.A.G., Klar N., Madrinan L.F., Olson L. (2013). Evaluating the effectiveness of road mitigation measures. Biodivers. Conserv..

[6] Benten A., Annighöfer P., Vor T. (2018). Wildlife warning reflectors’ potential to mitigate wildlife–vehicle collisions–a review on the evaluation methods. Front. Ecol. Evol..

[7] Stannard H.J., Wynan M.B., Wynan R.J., Dixon B.A., Mayadunnage S., Old J.M. (2021). Can virtual fences reduce wombat road mortalities?. Ecol. Eng..

[8] Umstatter C. (2011). The evolution of virtual fences: A review. Comput. Electron. Agric..

[9] Reeves J., Burnett S., Brunton E. (2021). Virtual fencing as a wildlife-vehicle collision mitigation measure: Technical function, wildlife response and considerations for installation in an urban environment. Aust. Zool..

[10] Kasbaoui N., Cooper J., Mills D.S., Burman O. (2016). Effects of long-term exposure to an electronic containment system on the behaviour and welfare of domestic cats. PLoS ONE.

[11] Masson S., de la Vega S., Gazzano A., Mariti C., Pereira G.D.G., Halsberghe C., Leyvraz A.M., McPeake K., Schoening B. (2018). Electronic training devices: Discussion on the pros and cons of their use in dogs as a basis for the position statement of the European Society of Veterinary Clinical Ethology. J. Vet. Behav..

[12] Kearton T., Marini D., Cowley F., Belson S., Lee C. (2019). The effect of virtual fencing stimuli on stress responses and behavior in sheep. Animals.

[13] Campbell D.L.M., Haynes S.J., Lea J.M., Farrer W.J., Lee C. (2019). Temporary exclusion of cattle from a riparian zone using virtual fencing technology. Animals.

[14] Ujvári M., Baagøe H.J., Madsen A.B. (1998). Effectiveness of wildlife warning reflectors in reducing deer–vehicle collisions: A behavioral study. J. Wildl. Manag..

[15] Brieger F., Hagen R., Vetter D., Dormann C.F., Storch I. (2016). Effectiveness of light-reflecting devices: A systematic reanalysis of animal–vehicle collision data. Accid. Anal. Prev..

[16] Benten A., Hothorn T., Vor T., Ammer C. (2018). Wildlife warning reflectors do not mitigate wildlife–vehicle collisions on roads. Accid. Anal. Prev..

[17] Ujvári M., Baagøe H.J., Madsen A.B. (2004). Effectiveness of acoustic road markings in reducing deer–vehicle collisions: A behavioural study. Wildl. Biol..

[18] Valitzski S.A., D’Angelo G.J., Gallagher G.R., Osborn D.A., Miller K.V., Warren R.J. (2009). Deer responses to sounds from a vehicle-mounted sound-production system. J. Wildl. Manag..

[19] Bender H., Coulson G. Roo the day: Evaluating the ShuRoo for prevention of macropod-vehicle collisions. Aust. Zool..

[20] Langbein J., Putman R.J., Pokorny B., Putman R.J., Apollonio M., Andersen R. (2011). Traffic collisions involving deer and other ungulates in Europe. Ungulate Management in Europe: Problems and Practice.

[21] D’Angelo G., van der Ree R., van der Ree R., Smith D.J., Grilo C. (2015). Use of reflectors and auditory deterrents to prevent wildlife–vehicle collisions. Handbook of Road Ecology.

[22] Fox S., Potts J.M., Pemberton D., Crosswell D. (2018). Roadkill mitigation: Trialing virtual fence devices on the west coast of Tasmania. Aust. Mammal..

[23] Coulson G., Bender H. (2019). Roadkill mitigation is paved with good intentions: A critique of Fox et al. (2019). Aust. Mammal..

[24] Fox S., Potts J. (2019). Virtual fence devices—A promising innovation: A response to Coulson and Bender (2019). Aust. Mammal..

[25] Englefield B., Candy S.G., Starling M.M., Paul D. (2019). A trial of a solar-powered, cooperative sensor/actuator, opto-acoustical, virtual road-fence to mitigate roadkill in Tasmania, Australia. Animals.

[26] Gunson K.E., Mountrakis G., Quackenbush L.J. (2011). Spatial wildlife-vehicle collision models: A review of current work and its application to transportation mitigation projects. J. Environ. Manag..

[27] Ramp D.J., Caldwell J., Edwards K.A., Warton D., Croft D.B. (2005). Modelling of wildlife fatality hotspots along the snowy mountain highway in New South Wales, Australia. Biol. Cons..

[28] Roger E., Bino G., Ramp D. (2012). Linking habitat suitability and road mortalities across geographic ranges. Landsc. Ecol..

[29] Skelton C.J., Cook A.S., West P., Spencer R.-J., Old J.M. (2019). Building an army of wombat warriors: Developing and sustaining a citizen science project. Aust. Mammal..

[30] Zandbergen P.A., Barbeau S.J. (2011). Positional accuracy of assisted GPS data from high-sensitivity GPS-enabled mobile phones. J. Navig..

[31] Merry K., Bettinger P. (2019). Smartphone GPS accuracy study in an urban environment. PLoS ONE.

[32] Parrish J.K., Burgess H., Weltzin J.F., Fortson L., Wiggins A., Simmons B. (2018). Exposing the science in citizen science: Fitness to purpose and intentional design. Integr. Comp. Biol..

[33] Downs R.R., Ramapriyan H.K., Peng G., Wei Y. (2021). Perspectives on citizen science data quality. Front. Clim..

[34] Matthews A., Green K. (2012). Seasonal and altitudinal influences on the home range and movements of common wombats in the Australian Snowy Mountains. J. Zool..

[35] Wrapson W., Harré N., Murrell P. (2006). Reductions in driver speed using posted feedback of speeding information: Social comparison or implied surveillance?. Accid. Anal. Prev..

[36] Jaarsma C.F., van Langevelde F., Botma H. (2006). Flattened fauna and mitigation: Traffic victims related to road, traffic, vehicle, and species characteristics. Transp. Res. D Transp. Environ..

[37] Visintin C., van der Ree R., McCarthy M.A. (2017). Consistent patterns of vehicle collision risk for six mammal species. J. Environ. Manag..

[38] Ascensão F., Yogui D., Alves M., Medici E.P., Desbiez A. (2019). Predicting spatiotemporal patterns of road mortality for medium-large mammals. J. Environ. Manag..

[39] Fabrizio M., Di Febbraro M., D’Amico M., Frate L., Roscioni F., Loy A. (2019). Habitat suitability vs landscape connectivity determining roadkill risk at a regional scale: A case study on European badger (*Meles mel**es*). Eur. J. Wildl. Res..

[40] Hobday A.J., Minstrell M.L. (2008). Distribution and abundance of roadkill on Tasmanian highways: Human management options. Wildl. Res..

[41] Rendall A.R., Webb V., Sutherland D.R., White J.G., Renwick L., Cooke R. (2021). Where wildlife and traffic collide: Roadkill rates change through time in a wildlife-tourism hotspot. Glob. Ecol. Conserv..

[42] Hobday A.J. (2010). Nighttime driver detection distances for Tasmanian fauna: Informing speed limits to reduce roadkill. Wildl. Res..

